# Increased Female MS Incidence and Differences in Gender-Specific Risk in Medium- and High-Risk Regions in Finland from 1981–2010

**DOI:** 10.1155/2013/182516

**Published:** 2013-11-10

**Authors:** Markus Holmberg, Annukka Murtonen, Irina Elovaara, Marja-Liisa Sumelahti

**Affiliations:** School of Medicine, University of Tampere, 33014 Tampere, Finland

## Abstract

*Background*. MS incidence has increased among females, suggesting the presence of environmental effect. *Object*. Regional differences and temporal changes in gender-specific MS incidence were studied in Finland. *Methods*. Cases from Jan 1, 1981 to Dec 31, 2010 in Pirkanmaa, Seinäjoki and Vaasa districts were included. The standardized incidence rates (SIR), incidences per 10^5^ person years with 95% confidence intervals (CI), and female-to-male ratios (F/M) were determined by district. *Results*. 1617 cases were included. Compared to Pirkanmaa, the MS risk was 1.9-fold (95% CI: 1.7–2.0) greater in Seinäjoki and 1.2-fold (95% CI: 1.1–1.4) in Vaasa, and the risk was high for both genders. The incidence trend stabilized in Seinäjoki and Vaasa, accompanied by an increase in the F/M ratio. A steady increase in Pirkanmaa was accompanied by a high F/M ratio. *Conclusion*. A high female preponderance accompanied a general increase in incidence since the 1990s, suggesting the influence of environmental factors. In high-risk districts, increased MS risk prevailed in both genders. High risk reflects both genetic and environmental effects. These effects may be shared with autoimmune diseases such as type 1 diabetes mellitus; the incidence of which follows MS in Finland. Population-based case-control studies are needed to identify these factor effects.

## 1. Introduction

MS incidence has increased, particularly among females [1–4], indicating the influence of environmental factors. The integration of magnetic resonance imaging (MRI) in diagnostic criteria since the 1990s and progress in immunomodulatory drug treatments have contributed to these increasing rates [5, 6]. 

Epidemiologically, MS is characterized by an uneven geographical distribution [7]. Studies performed since 1964 confirm this observation in Finland [8–10], which is located in Northern Europe between the latitudes 60 and 70°N. High-risk areas in the western districts, Seinäjoki and Vaasa, are characterized by an irregular incidence pattern, and an increased male risk was observed in Seinäjoki in 1979–1993 [10]. 

We aimed to analyze the gender-specific incidence in high- and medium-risk areas in 1981–2010 to make inferences on the etiological factors correlated with high-risk groups. The incidence in the former medium-risk area Pirkanmaa [8] is studied for the first time. Incidence is regarded as the most important indicator of disease frequency, and changes in incidence reflect environmental factors in genetically stable populations. 

## 2. Materials and Methods

**Figure 1:**
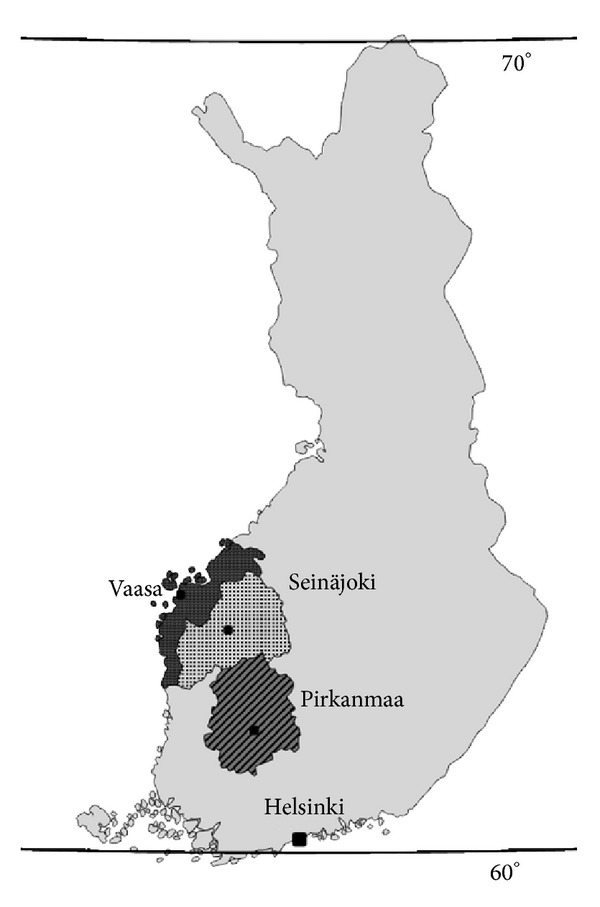
Map of Finland. Finland lies in Scandinavia, North Europe between latitudes 60–70°N. Gulf of Bothnia in northern most part of Baltic Sea limits the west coast. The total population was 5.4 million in 2010. The University Hospital District of Tampere is demarcated, and location of central hospitals and hospital districts in Pirkanmaa (oblique lines), Seinäjoki (dots), and Vaasa (dark grey) are pointed in the map.

The districts examined in this study are shown in Figure 1. The total population in 2010 was 850630 [11]. The Pirkanmaa Central Hospital (population 485911) is one of five University hospitals in Finland. The Seinäjoki and Vaasa Central Hospitals serve populations of 198469 and 166250, respectively [11]. Both Seinäjoki and Vaasa are mainly rural districts, while Pirkanmaa is more urbanized, which is reflected in the age-structure and distribution of its populations.

From 1981 to 2010, the population increased by 19% in Pirkanmaa and 9% in Vaasa, while a 2% decrease was reported in Seinäjoki. A 21% decrease in the age group 0–39 years was observed in Seinäjoki and Vaasa [11]. 

Neurological services were evenly distributed between the district hospitals. MS is diagnosed by neurologists in central or university hospitals in Finland [10]. CSF is a routine examination in MS diagnostics. MRI scans have been used in diagnostics from 1990 in Pirkanmaa and 1993 in Seinäjoki and Vaasa.

The National Institute for Health and Welfare and the local ethical standards committee approved the retrospective examination of identified patient records in the hospitals in this study.

Patients with multiple sclerosis in the health care districts of Pirkanmaa, Seinäjoki and Vaasa, including Jacobstad, were recruited from hospital registries from January 1, 1981 to December 31, 2010 according to multiple sclerosis or morbus demyelinans diagnoses and optic- or retrobulbar neuritis (340, 341, and 377 in the *International Classification of Diseases*, ICD versions 8 and 9, G35, G37, and H46 in ICD-10). The patient records were then examined by the authors (Markus Holmberg, Annukka Murtonen, and Marja-Liisa Sumelahti). Patient information was collected on the date, and the quality of the first symptoms and clinical findings were recorded. Confirmatory positive results at the time of diagnosis were obtained from patient records for MRI, cerebrospinal fluid (CSF), and evoked potentials (EP). The dates of the follow-up MRI results were also recorded. Patient cases were included in the analyses when fulfilling the criteria of clinically (CD) or laboratory supported definite (LSD) MS as previously described by Poser [12] during January 1, 1981 to December 31, 2010, and when the patients resided in the study districts at the year of diagnosis. The patient's residence was updated using a personalized identification number and the year of diagnosis at Statistics Finland.

The age-adjusted and gender-specific incidence per 10^5^ person-years was calculated from January 1, 1981 to December 31, 2010 for three 10-year-periods (1981–1990, 1991–2000, and 2001–2010) with a 95% confidence interval (CI) by district. The incidence of the 5-year-periods did not change our overall conclusions (data not shown). Furthermore, to avoid chance variation, we used 10-year-periods in our calculations. Due to the small number of cases in the age-specific strata, indirect standardization [13] was used to study regional risk during 1981–2010. In this study, the standard population used was the Pirkanmaa population. The incidence rates in Pirkanmaa from 1981 to 2010 in each 10-year-age group were used to calculate the expected numbers of cases for both the Seinäjoki and Vaasa populations. The resulting expected number of cases was then compared to the actual observed numbers of cases in Seinäjoki and Vaasa, resulting in the standardized incidence rate (SIR), which was the ratio of observed and expected cases. The total and gender-specific SIRs with a 95% CI are presented.

## 3. Results

**Figure 2:**
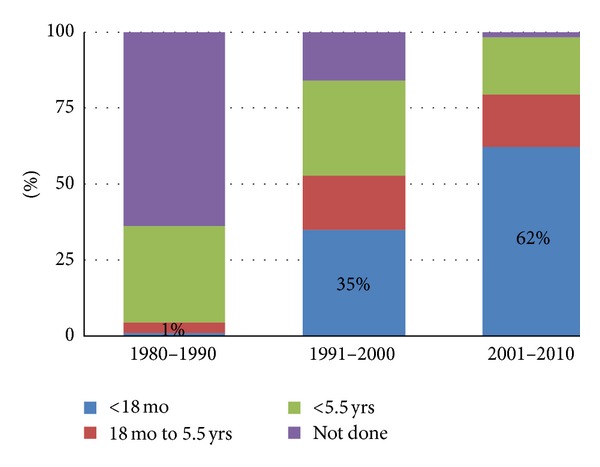
Percentage of performed MRI scans by time from disease onset among MS cases diagnosed between 1981 and 2010.

The patient characteristics of 1617 cases is shown in Table 1. The onset symptoms were evenly distributed (the district-specific figures are not shown), as were the total F/M ratio (2.2), median age at onset (32.0), and age at diagnosis (37.0 years). The median diagnostic delay decreased from 4.0 years to 2.0 years (Chi-square test *P* < 0.001) during the study periods. Paraclinical studies in 10-year-periods are shown in Table 2. The CSF (including either the IgG index, immunoelectrophoresis, or both) was performed in 89–92% of the cases. The decreasing use of evoked potentials (27% to 19%) and the increasing use of MRI (36% to 98%) was observed during the follow-up evaluation. The second scan, which was used to study dissemination in time and space in 65% of the cases, was performed during the next 18 months after the first scan in 58% of the cases, 94% of which were positive for MS. The delay to the first MRI from the date of onset of symptoms was significantly decreased during the follow-up evaluation (Figure 2).

From January 1, 1981 to December 31, 2010, the age-adjusted incidence was 6.7 × 10^5^ (95% CI: 6.2–7.2) in Pirkanmaa, 12.5 × 10^5^ (95% CI: 11.5–13.5) in Seinäjoki, and 8.3 × 10^5^ (95% CI: 7.4–9.2) in Vaasa. The SIR was 1.9 (95% CI: 1.7–2.0) in Seinäjoki and 1.2 (95% CI: 1.1–1.4) in Vaasa, compared to Pirkanmaa (SIR 1.0).

The incidence for 10-year-periods (1981–1990, 1991–2000, and 2001–2010) is shown in Table 3. A steady increase in Pirkanmaa (from 5.1 to 8.2) and a two-fold increase in both Seinäjoki (from 7.1 to 14.7) and Vaasa (from 5.7 to 11.7) were observed. The female incidence was significantly higher in all age groups and in all districts. The male incidence decreased during the last decade in Vaasa and Seinäjoki. The F/M ratios remained stable in Pirkanmaa (2.5, 2.1, 2.2) and increased 1.3-fold (1.8, 1.9, and 2.3) in Seinäjoki and 1.6-fold (1.8, 2.1, and 2.8) in Vaasa.

**Figure 3:**
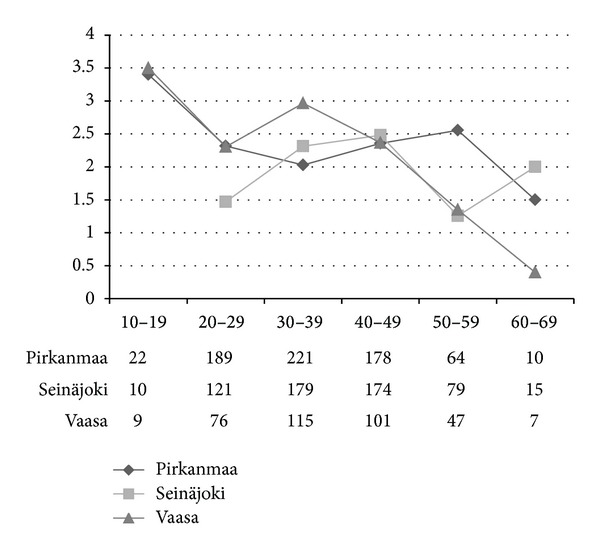
F/M-ratio by total number in 10-year-age groups among cases diagnosed during 1981–2010 in hospital districts of Pirkanmaa, Seinäjoki, and Vaasa. There were no men in the 10–19 group in Seinäjoki.

The F/M ratios in the 10-year-age groups according to district are shown in Figure 3. The highest F/M ratio of 3.5 was observed in the 10–19-year-age group. In Pirkanmaa, the ratio subsequently remained stable, whereas peaks were observed in Seinäjoki (age group: 30–49-years) and Vaasa (age group: 30–39-years).

**Figure 4:**
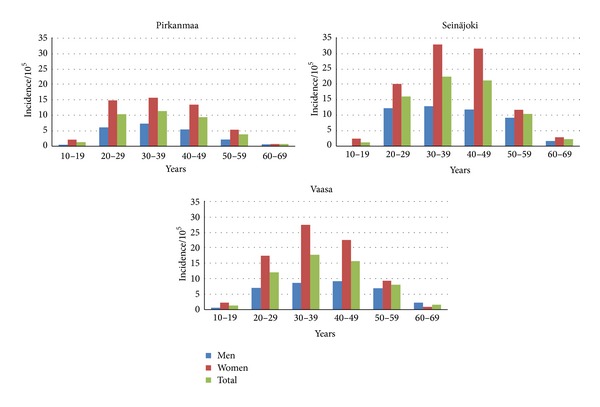
Incidence of MS in the 10-year-age groups in Pirkanmaa, Seinäjoki, and Vaasa hospital districts during the period 1981–2010.

Female risk values in Seinäjoki (SIR 1.8, 95% CI: 1.7–2.0) and Vaasa (1.5, 95% CI: 1.3–1.7) were similar and the age-specific incidence peaked at 30–39 years (Figure 4). Male risk in Seinäjoki was 2-fold compared to Pirkanmaa (SIR 2.00, 95% CI: 1.7–2.3) and the incidence was high in the large age group (20–59 year olds). In Vaasa, male risk (1.4, 95% CI: 1.2–1.7) peaked in the older age groups.

## 4. Discussion

Regional differences in MS epidemiology were among the earliest observations made in Finland [8–10]. Earlier observations on high-risk MS in Seinäjoki have also been confirmed here, because the 30-year-incidence rate of 12.5/10^5^ person-years in 2010 is the highest reported [8, 10]. The incidence was 8.3 and 6.7 in Vaasa and Pirkanmaa, respectively. However, the regional MS risk in Seinäjoki remains nearly two-fold (SIR 1.9) compared to Vaasa (1.2) and Pirkanmaa (1.0) in Finland. The southern and urbanized Uusimaa district, which locates the capital city Helsinki, and Pirkanmaa (formerly Häme) are regarded as regions with similar and standard MS risk in Finland [8, 10]. This perception holds true, because an incidence of 5.1 in Uusimaa was reported in 1993 [10], which was similar to the rate of 5.1 in Pirkanmaa in 1981–1990. 

Consistent with these findings, contemporary studies performed in Ireland and Wales [2, 14] revealed an approximately 1.5-fold increase in the F/M ratio, observed also in this study in districts of Seinäjoki and Vaasa from 1981–2010. Ratios of 2.3 in Seinäjoki and 2.8 in Vaasa in 2010 exceeded the corresponding ratios of 1.6 and 2.2 in an earlier study from 1979–1993 [10]. However, the ratio in Pirkanmaa remained stable, which was consistent with other previous studies [15, 16]. 

MS affects women during their most active years of life. In this study, we showed the highest F/M ratio of 3.5 in the 10–19-year-age group, where the total incidence remains low. The female preponderance remained high in the 20–59-year-age group. Thus, the high F/M ratio in the youngest age group may indicate the presence of risk factors affecting this female subpopulation, independent of a geographically associated risk. 

The local risk of multiple sclerosis in Seinäjoki may be explained by genetic factors because HLA characterization has demonstrated increased frequencies for B7, B12, and DR2 among both patients and their healthy relatives [17]. The patients' families were subsequently examined for the myelin basic protein (MBP) gene on chromosome 18, which is a candidate gene involved in multiple sclerosis. Genetic linkage and association analyses suggested that a genetic predisposition to multiple sclerosis was closely linked to the MBP gene in this population [18]. Despite this finding, the genetic background was similar in rural areas of partially Swedish-speaking Vaasa, Finnish-speaking Seinäjoki, and the more urbanized Pirkanmaa [19–21]. 

Risk among males was increased in Seinäjoki despite the presence of a declining male incidence. The SIR for males remained 2-fold (2.0, 95% CI: 1.7–2.3), with an SIR of 2.8 (95% CI: 2.3–3.5) reported in an earlier study in 1979-93 [10]. Although the SIRs for males in both Seinäjoki and Vaasa (1.4) were higher compared to Pirkanmaa, they did not differ from the female ratios. Given that the populations examined were genetically homogeneous and stable and that the genetic changes in these populations were slow, the gender-specific incidence trends observed in this study indicated that environmental factors affecting the increased male MS risk in high-risk districts were less powerful than factors affecting the increasing female preponderance in the 1990s. According to this observation, the factors affecting males and females may be different in general, and some of these factors may act temporally and locally.

The common awareness of MS and the availability of MRI from 1993 in all districts, together with revised criteria and justified early DMT start in RRMS have also precipitated MS diagnosis in Finland [22, 23]. The diagnostic evaluation performed in this study was on the basis of the International and National Current Care Guidelines [24–26]. Due to a highly centralized health care system, we were able to obtain full coverage of the MS patients in this population-based study. The diagnosis-based incidence was used to avoid proportional incidence and underestimated risk, in addition to problems with recognition and the definition of the historical and often arbitrary onset symptoms in long-term follow-up evaluations [10]. We included definite MS cases to consolidate regional and temporal comparisons. Active CSF sampling in Finland promoted the application of Poser criteria [12], in which a positive finding is essential. CSF s considered fundamental in differential diagnostics for MS [27]. Because purely clinical diagnoses were nearly nonexistent in 2001–2010, diagnostic specificity may be considered high in this cohort. However, the number of cases was fairly low, and the population in central parts of the country was younger compared to Seinäjoki and Vaasa, which was among the factors that may confound a regional and temporal comparison of incidence [11]. Thus, because small numbers were generally present in the age- and gender-specific strata, we decided to use indirect standardization in the risk calculations performed in this study [13]. 

We observed a significant decrease in the diagnostic delay, which was consistent with rapid MR imaging after the first symptoms in 2001–2010 in each district. Despite this finding, the incidence during the last ten-year period in both Vaasa and Seinäjoki showed a stabilization after a two-fold increase in 1981–2000. In 1981–2010, the population at risk in the 10–39-year-age groups decreased to 11% and 26% in Vaasa and Seinäjoki, respectively. The number of MS cases in 2001–2010 decreased by 21% in both Seinäjoki (a decrease from 122 to 96 patients) and Vaasa (from 80 to 63 patients), which was observed in males and caused the recent stabilization and observed incidence decline. In Pirkanmaa, the population only decreased by 3% and the number of new MS cases increased by 23% (from 130 to 168 patients), which was accompanied by a steadily increasing trend. Despite these recent differences in population structure, the current sources of livelihood were similar in the districts, including those of the Swedish-speaking population (22%) in the Vaasa district, which may indicate that the environmental risk factors may be connected to changes and differences in lifestyle.

In conclusion, Seinäjoki and Vaasa represented high MS risk areas, where risk was observed to be high among females, peaking in the youngest age group, and among males in a large age group. However, high risk in general reflects both genetic and environmental effects. These effects may also be shared among other autoimmune diseases, such as type 1 diabetes mellitus, which predominantly affects males. The incidence of type 1 diabetes mellitus closely follows MS, both geographically and temporally, in Finland [28]. Thus, population-based case control studies are required to further characterize these factor effects, which may include a role for Vitamin D, several lifestyle factors (including smoking, obesity, and hormone replacement therapy), and later childbirth among females.

## Figures and Tables

**Table 1:** Characteristics of MS cases in Pirkanmaa, Seinäjoki, and Vaasa during the period 1981–2010.

Number of cases, *n* (%)	
Total	1617
Male	512 (32)
Female	1105 (68)
F/M	2.2
Diagnosis using Poser, *n* (%)	
CDMS	60 (4)
CDMS + paraclinical	1223 (75)
LSDMS	75 (5)
LSDMS + paraclinical	259 (16)
Age, years	
At onset, median	32
At the time of diagnosis, median	37
Diagnostic delay, years	
Mean	4.6
Median	2.0
First symptoms by anatomic level, %	
Brainstem/cerebellum	25
Visual tract	22
Sensory	22
Medullary	19
Pyramidal tract above medulla	5
Other/UN	4
Multiple	3

**Table 2:** Paraclinical test results among cases with definite MS diagnosis by 10-year periods from 1981–2010.

Hospital district	1981–1990				1991–2000				2001–2010			
	Cases	CSF	EP	MRI	Cases	CSF	EP	MRI	Cases	CSF	EP	MRI
*Pirkanmaa *	167	152	88	72	219	209	98	199	298	282	50	292
Percentage performed		91%	53%	43%		95%	45%	91%		95%	17%	98%
Positive findings (%)		150 (99%)	73 (83%)	67 (93%)		192 (92%)	79 (81%)	176 (88%)		255 (90%)	38 (76%)	267 (91%)
*Seinäjoki *	123	106	7	36	237	215	69	189	218	200	70	213
Percentage performed		86%	6%	29%		91%	29%	80%		92%	32%	98%
Positive findings (%)		97 (92%)	5 (71%)	32 (89%)		194 (90%)	48 (70%)	176 (93%)		173 (87%)	50 (71%)	196 (92%)
*Vaasa *	70	63	1	21	142	126	9	117	143	126	6	140
Percentage performed		90%	1%	30%		89%	6%	82%		88%	4%	98%
Positive findings (%)		55 (87%)	1 (100%)	21 (100%)		103 (82%)	6 (67%)	109 (93%)		102 (81%)	5 (83%)	129 (92%)

*Total *	360	321	96	129	598	550	176	505	659	608	126	645
Percentage performed		89%	27%	36%		92%	29%	84%		92%	19%	98%
Positive findings (%)		302 (94%)	79 (82%)	120 (93%)		489 (89%)	133 (76%)	461 (91%)		530 (87%)	93 (74%)	592 (92%)

CFS: cerebrospinal fluid, Ep: evoked potential, and MRI: magnetic resonance imaging.

**Table 3:** Incidence of MS during the three decades examined in Pirkanmaa, Seinäjoki, and Vaasa.

Period	1981–1990			1991–2000			2001–2010		
District	Inc/10^5^	95% CI	*N *	Inc/10^5^	95% CI	*N *	Inc/10^5^	95% CI	*N *
Pirkanmaa									
Males	3.0	(2.2–3.8)	48	4.1	(3.1–5.1)	70	5.1	(4.1–6.1)	92
Females	7.1	(5.8–8.4)	119	8.8	(7.4–10.2)	149	11.5	(9.9–13.1)	206
Total	5.1	(4.3–5.9)	167	6.5	(5.4–7.4)	219	8.2	(7.3–9.1)	298
Seinäjoki									
Males	5.0	(3.5–6.5)	44	10.5	(8.2–12.8)	83	8.6	(6.5–10.7)	66
Females	9.2	(7.2–11.2)	79	20.3	(17.1–23.5)	154	21.0	(17.7–24.3)	152
Total	7.1	(5.8–8.4)	123	15.3	(13.4–17.2)	237	14.7	(12.7–16.7)	218
Vaasa									
Males	4.1	(2.5–5.7)	25	7.4	(6.3–8.5)	46	6.0	(4.6–7.4)	38
Females	7.4	(5.2–9.6)	45	16.0	(14.4–17.6)	96	17.6	(14.2–21.0)	105
Total	5.7	(4.4–7.1)	70	11.7	(10.7–12.7)	142	11.7	(9.8–13.6)	143

## References

[1] Koch-Henriksen N, Sørensen PS (2010). The changing demographic pattern of multiple sclerosis epidemiology. *The Lancet Neurology*.

[2] Gray OM, McDonnell GV, Hawkins SA (2008). Factors in the rising prevalence of multiple sclerosis in the North-East of Ireland. *Multiple Sclerosis*.

[3] Simpson S, Blizzard L, Otahal P, van der Mei I, Taylor B (2011). Latitude is significantly associated with the prevalence of multiple sclerosis: a meta-analysis. *Journal of Neurology, Neurosurgery and Psychiatry*.

[4] Alonso A, Hernán MA (2008). Temporal trends in the incidence of multiple sclerosis: a systematic review. *Neurology*.

[5] Tintoré M, Rovira A, Río J (2003). New diagnostic criteria for multiple sclerosis: application in first demyelinating episode. *Neurology*.

[6] Filippi M, Rocca MA (2011). MR imaging of multiple sclerosis. *Radiology*.

[7] Compston A (1997). Genetic epidemiology of multiple sclerosis. *Journal of Neurology Neurosurgery and Psychiatry*.

[8] Wikström J (1975). Studies on the clustering of multiple sclerosis in Finland. II: microepidemiology in one high risk county with special reference to familial cases. *Acta Neurologica Scandinavica*.

[9] Kinnunen E (1984). Multiple sclerosis in Finland: evidence of increasing frequency and uneven geographic distribution. *Neurology*.

[10] Sumelahti ML, Tienari PJ, Wikström J, Palo J, Hakama M (2000). Regional and temporal variation in the incidence of multiple sclerosis in Finland 1979–1993. *Neuroepidemiology*.

[11] Statistics Finland http://www.stat.fi/tup/suoluk/suoluk_vaesto.html#bruttokansantuote.

[12] Poser CM, Paty DW, Scheinberg L (1983). New diagnostic criteria for multiple sclerosis: guidelines for research protocols. *Annals of Neurology*.

[13] Armitage P, Berry G, Armitage P, Berry G (1994). Standardization. *Statistical Methods in Medical Research*.

[14] Hirst C, Ingram G, Pickersgill T, Swingler R, Compston DAS, Robertson NP (2009). Increasing prevalence and incidence of multiple sclerosis in South East Wales. *Journal of Neurology, Neurosurgery and Psychiatry*.

[15] Orton SM, Herrera BM, Yee IM (2006). Sex ratio of multiple sclerosis in Canada: a longitudinal study. *The Lancet Neurology*.

[16] Celius EG, Smestad C (2009). Change in sex ratio, disease course and age at diagnosis in Oslo MS patients through seven decades. *Acta Neurologica Scandinavica*.

[17] Kinnunen E, Koskimies S, Lagerstedt A, Wikstrom J (1984). Histocompatibility antigens in familial multiple sclerosis in a high-risk area of the disease. *Journal of the Neurological Sciences*.

[18] Tienari PJ, Wikström J, Sajantila A, Palo J, Peltonen L (1992). Genetic susceptibility to multiple sclerosis linked to myelin basic protein gene. *The Lancet*.

[19] Nevanlinna HR (1972). The Finnish population structure. A genetic and genealogical study. *Hereditas*.

[20] Salmela E, Lappalainen T, Fransson I (2008). Genome-wide analysis of single nucleotide polymorphisms uncovers population structure in Northern Europe. *PLoS ONE*.

[21] Hannelius U, Salmela E, Lappalainen T (2008). Population substructure in Finland and Sweden revealed by the use of spatial coordinates and a small number of unlinked autosomal SNPs. *BMC Genetics*.

[22] McDonald WI, Compston A, Edan G (2001). Recommended diagnostic criteria for multiple sclerosis: guidelines from the international panel on the diagnosis of multiple sclerosis. *Annals of Neurology*.

[23] Polman CH, Reingold SC, Edan G (2005). Diagnostic criteria for multiple sclerosis: 2005 revisions to the ‘McDonald criteria’. *Annals of Neurology*.

[24] Goodin DS, Frohman EM, Garmany GP (2002). Disease modifying therapies in multiple sclerosis: report of the therapeutics and technology assessment subcommittee of the American academy of neurology and the MS council for clinical practice guidelines. *Neurology*.

[25] NICE guideline for management of multiple sclerosis. http://www.nice.org/.

[26] Working Group Appointed by the Finnish Medical Society Duodecim and the Finnish Neurological Society Current care guideline: multiple sclerosis.

[27] Freedman MS, Thompson EJ, Deisenhammer F (2005). Recommended standard of cerebrospinal fluid analysis in the diagnosis of multiple sclerosis: a consensus statement. *Archives of Neurology*.

[28] Soltesz G, Patterson CC, Dahlquist G (2007). Worldwide childhood type 1 diabetes incidence—what can we learn from epidemiology?. *Pediatric Diabetes*.

